# Successful strategies for high participation in three regional healthcare surveys: an observational study

**DOI:** 10.1186/1471-2288-11-176

**Published:** 2011-12-30

**Authors:** Kristen R Elkins, Christopher M Nguyen, Diane S Kim, Hildy Meyers, Michele Cheung, Susan S Huang

**Affiliations:** 1 Division of Infectious Diseases and Health Policy Research Institute, University of California, Irvine School of Medicine, Orange, California, USA; 2 Department of Epidemiology and Assessment, Orange County Healthcare Agency, Santa Ana, California, USA

## Abstract

**Background:**

Regional healthcare facility surveys to quantitatively assess nosocomial infection rates are important for confirming standardized data collection and assessing health outcomes in the era of mandatory reporting. This is particularly important for the assessment of infection control policies and healthcare associated infection rates among hospitals. However, the success of such surveys depends upon high participation and representativeness of respondents.

**Methods:**

This descriptive paper provides methodologies that may have contributed to high participation in a series of administrative, infection control, and microbiology laboratory surveys of all 31 hospitals in a large southern California county. We also report 85% (N = 72) countywide participation in an administrative survey among nursing homes in this same area.

**Results:**

Using in-person recruitment, 48% of hospitals and nursing homes were recruited within one quarter, with 75% recruited within three quarters.

**Conclusions:**

Potentially useful strategies for successful recruitment included in-person recruitment, partnership with the local public health department, assurance of anonymity when presenting survey results, and provision of staff labor for the completion of detailed survey tables on the rates of healthcare associated pathogens. Data collection assistance was provided for three-fourths of surveys. High compliance quantitative regional surveys require substantial recruitment time and study staff support for high participation.

## Background

Regional and national evaluations of healthcare facilities are increasingly performed to provide local benchmarks for hospital and nursing home quality measures [1-8]. In the United States, many states have or are implementing mandatory reporting requirements for healthcare-associated infections [9].

The intent of mandatory reporting is to allow inter-facility comparisons; however, such comparisons require standardized definitions and data collection [5,6,10-13]. Regional surveys, particularly those including both qualitative and quantitative data collection, can help ascertain not only estimates of healthcare-associated infection outcomes, but whether data collection, definitions, and policies are uniform. These studies to evaluate whether the process is consistent across facilities are not commonly undertaken due to the scope of work, expense, limited staffing resources, and uncertain participation. When regional studies are performed, descriptions of the methodology are limited and little is learned or shared about successes and failures related to recruitment, implementation, and data acquisition [2,3,14,15].

We developed healthcare-associated infection surveys targeting all 31 hospitals and 72 nursing homes across a single county in order to assess facility-specific methods of collecting and reporting acquisition and infection rates due to highly antibiotic-resistant bacteria. We describe here the research strategies used to obtain high participation, develop and maintain strong participant relationships, and ensure comprehensive data collection from multiple data streams in a regional study of healthcare facilities. We further provide our interpretation of the relative value of specific strategies in contributing to high participation rates.

## Methods

### Surveys

We conducted three surveys in healthcare facilities across a Southern California metropolitan county of three million people (Orange County, California). These surveys were part of a larger project known as Project MAPP (Mapping and Analyzing Patient Pathways), a joint collaboration between investigators at the Health Policy Research Institute at the University of California Irvine School Of Medicine and the Orange County Health Care Agency, the county department of public health. The three surveys were aimed at the Health Information Management/Medical Records Departments (hospitals and nursing homes), Infection Control and Prevention Departments (hospitals only), and Microbiology Laboratories (hospitals only) of all 31 hospitals and 72 nursing homes in the county. One additional hospital was imminently closing and was not considered eligible. Surveys involved providing monthly facility-specific data on the incidence of healthcare-associated pathogens for a one-year period, as well as answering questions on facility characteristics, definitions used for routine surveillance of healthcare-associated pathogens, and infection control policies. Recruitment for participation occurred during a 15-month period in 2008 and 2009.

Each survey consisted of two parts, a multiple choice/short answer section and a portion involving tables requiring facility-level monthly data for a 12-month period. The Administrative Survey requested information on the volume and size of facilities, ward types, and fraction of total patients and patients known to harbor specific healthcare-associated pathogens who are admitted from and discharged to specific healthcare facilities within the county. It consisted of 5 questions and 4 data tables each for pediatric and adult patient populations. The Infection Control Survey posed descriptive questions about infection control and prevention practices and requested facility-level definitions and data tables related to nosocomial rates of methicillin-resistant *Staphylococcus aureus*, vancomycin-resistant enterococcus, *Clostridium difficile*, and highly resistant gram-negative pathogens. It consisted of 38 questions and 10 data tables each for pediatric and adult patient populations. The Microbiology Laboratory Survey requested information on routine microbiologic processing methods and facility specific data tables on all positive cultures of the same organisms listed above. It consisted of 7 multiple choice questions and 18 data tables. All surveys were accompanied by a detailed set of instructions. Since our focus in this paper is to describe the effectiveness of our strategies to recruit and retain participants for these surveys, we have provided the structure, but not the content, of these surveys, which will be described elsewhere. This study was jointly approved by the institutional review boards of the University of California Regents and the Orange County Health Care Agency.

### Preparatory Strategies

A formal partnership was forged with the Orange County Health Care Agency for the implementation of these surveys. This partnership created a strong association between this project and a trusted and familiar public health agency. It provided a strong written endorsement from the Orange County Health Care Agency and enabled use of the Orange County Health Care Agency logo on project materials. Study staff carried both University of California Irvine and Orange County Health Care Agency badges.

Local chapters of healthcare and infection control and prevention societies were contacted and engaged to garner project support and visibility. Presentations were given to the local chapters of the Association for Professionals in Infection Control and Epidemiology and the California Association of Health Facilities (an association of nursing homes). Active members of these groups included key contact individuals at several hospitals and nursing homes for both the Administrative Survey and Infection Control Survey. In addition, we provided Medicine Grand Rounds presentations to local hospitals on the topic of methicillin-resistant *Staphylococcus aureus *and introduced Project MAPP at those forums.

The Project MAPP name and logo were placed on all study materials, including survey instruments, letterhead, slide presentations, and business cards. All surveys were printed in color and presented in multi-sectioned presentation binders. All staff wore business attire for all engagements.

Study staff were instructed to keep detailed prospective notes about all their recruitment encounters, facility-specific issues, and methods for garnering and maintaining participation

### Recruitment Strategies

A core group of six advisors representing members of Infection Control and Prevention programs in Orange County were asked to provide feedback on survey design, survey content, and methods for recruitment. Advisors included representatives from both pediatric and adult hospitals, the Orange County Health Care Agency, as well the current president from the local Association for Professionals in Infection Control and Epidemiology chapter.

In all cases, recruitment occurred through scheduled face-to-face meetings at which all survey elements were discussed and reviewed. Study staff contact information was provided with all surveys, and follow-up phone calls and visits were routinely performed. When formal inter-facility relationships existed (e.g., jointly-owned facilities, multi-facility corporations), recruitment was coordinated so as to solicit corporate approval or to enable leadership from one facility to encourage participation by all related facilities.

Verbal and written assurances of each facility's confidentiality were given. That is, a reassurance to prospective participants that no facility names would be disclosed in association with collected data, and those data would be reported and published only in aggregate form with other participating facilities. All participants were told they would be given a report at the end of the study that displayed their performance compared to summary level results from all county participants. Participants were free to share reports amongst themselves at their own discretion.

In addition to providing feedback of countywide results, token gifts were offered for completing the surveys (estimated at 4-10 hours not including staff labor-see below). All gifts were valued to express appreciation (approximately $50-250, based upon required effort), but were not sufficient to repay participation time. Administrative Survey gifts were primarily food gift baskets; Infection Control Survey gift options included food gift baskets, infection control reference books, and infection control-related teaching items; Microbiology Laboratory Survey gift options included food gift baskets, microbiology reference books, and microbiology-related items.

### Data Collection Strategies

Once participation was garnered, a high level of persistence was used to promote consistent and timely receipt of the completed surveys (e.g., interval phone calls, in-person visits, and follow-up e-mails). During this period, facilities were generally contacted at least twice per month. In all instances, research staff offered to assist with data collection. If labor was requested, project staff complied with each facility's regulations for viewing identifiable data, even though only summary level counts were recorded and retained. All surveys required the completion of data tables covering a recent 12-month period.

### Analysis

This was a descriptive analysis. Strategies implemented during preparation, recruitment, and data collection are described according to their perceived effectiveness by study staff. Obstacles encountered during the project were also described along with the strategies used to successfully overcome them. Project success was measured by the percentage of participating facilities, time required for recruitment and survey completion, completeness of data returned, and the amount of effort required by project staff. We evaluated the time to recruitment and time to survey completion in quarters, and described specific details that affected participation or data collection. We categorized received surveys as 25%,50%,75%, or 100% complete, and reported the fraction of surveys that fell into each of these categories. In addition, we calculated the fraction of facilities requiring varying amounts of study staff labor (categorized as little to none, some, or extensive) to complete the surveys. Finally, full-time study staff were asked to independently rate the value of specific strategies during the following stages of the project - Preparation, Recruitment, and Data Collection. We used a rating scale of 1 to 4, with 1 being unimportant, 2 being somewhat important, 3 being very important, and 4 being critical to success. Average ratings were calculated.

Characteristics of Orange County hospitals were obtained from California state publicly available resources [16].

## Results

### Participation

All Orange County hospitals(N = 31) participated in at least one survey, including six small long term acute care facilities that provided chronic medical care such as ventilator support. Characteristics of the 31 hospitals are found in Table 1.

**Table 1:** Patient Characteristics of 31 Orange County Hospitals, 2007

Hospital Characteristic	Median (Range) Among Orange County Hospitals
Licensed Beds	178 beds (27-505)
Acute Care Hospitals (N = 26)	202 beds (48-505)
Long-term Acute Care Hospitals (N = 6)	85 beds (27-177)
Annual Admissions	8,768 (101-32,931)
Acute Care Hospitals	11,178 (2,385-32,931)
Long-term Acute Care Hospitals	1,001 (101-4,182)
Median Age in Years	
Adult ^a^	55 yrs (35-77)
Pediatric ^b, c^	15 yrs (2-17)
Male	40% (33-59)
Race	
White	77% (19-92)
Black	2% (1-18)
Asian	8% (0-44)
Other	8% (0-72)
Unknown	1% (0-16)
Hispanic Ethnicity	21% (5-77)
Average Length of Stay	
Adult ^a^	5.4 days (3.9-33.2)
Acute Care Hospitals	5.0 days (3.9-6.9)
Long-term Acute Care Hospitals	12.3 days (4.2-33.2)
Pediatric ^b^	3.9 days (2.5-31.6)
Acute Care Hospitals	3.6 days (2.5-18.3)
Long-term Acute Care Hospitals	20.7 days (9.7-31.6)
Insurer Type	
Medicare	32% (0-88)
Medicaid	16% (2-66)
Private Coverage	34% (6-85)

^a ^Adult calculation completed for patients ≥ 18 years at Adult Hospitals (n = 29)

^b ^Pediatric calculation completed for patients at Children's Hospitals (n = 3) and Adult Hospitals with > 100 pediatric visits (n = 22)

^c ^Newborns excluded

Overall 29(94%) and 27(87%) hospitals agreed to participated in the Administrative and Infection Control Surveys, respectively. All 16 eligible Orange County microbiology laboratories agreed to participate in the Microbiology Laboratory Survey, representing 31 hospitals. Of those facilities that agreed to participate 27/29 Administrative surveys, 26/27 Infection Control surveys, and 13/16 Microbiology Laboratory surveys were received. Nursing homes were recruited for only the Administrative Survey, and among the 72 countywide nursing homes, 65(90%) agreed to participate, and 61 surveys were received.

### Preparatory Strategies for High Participation

**Figure 1:**
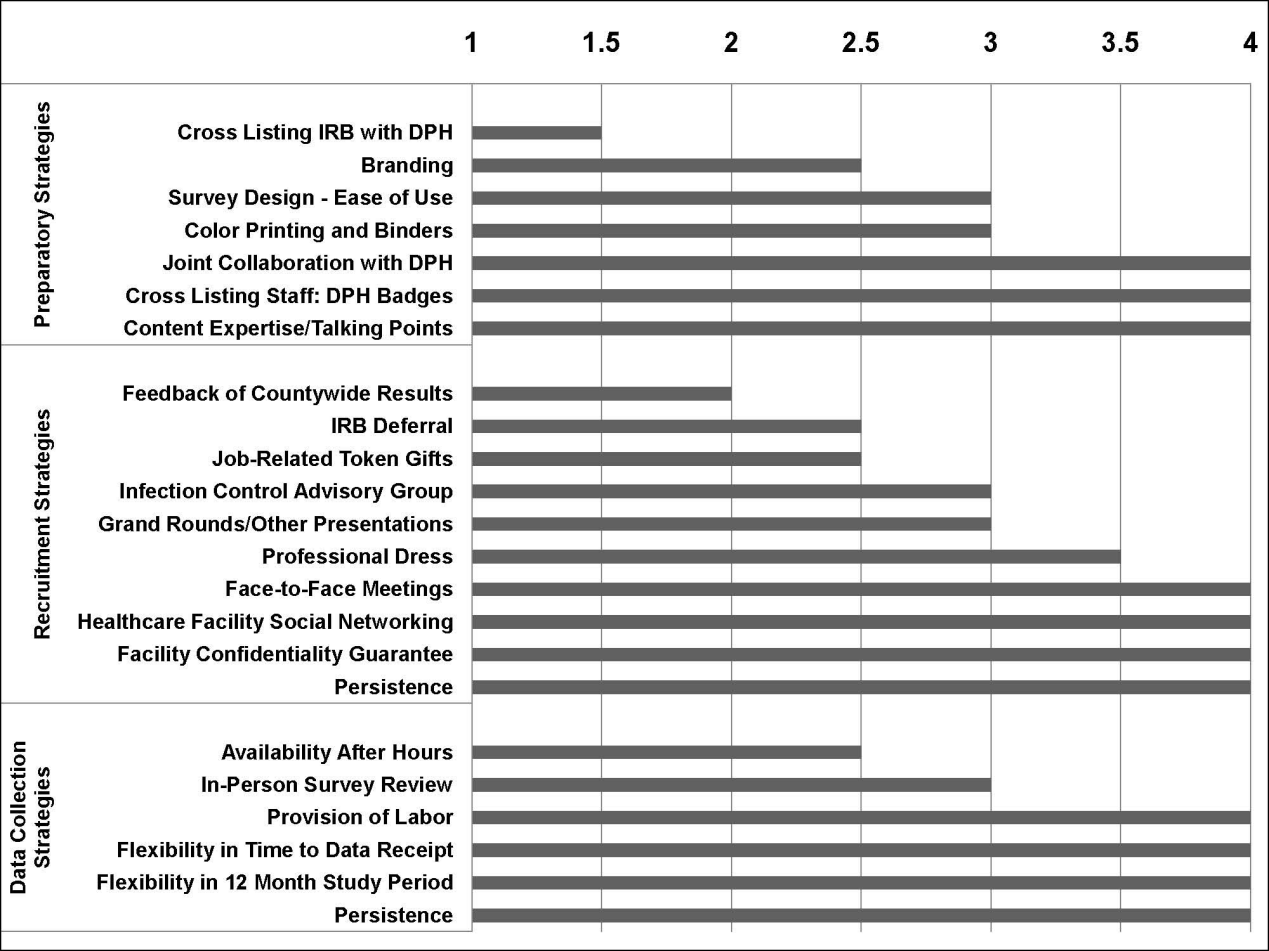
**Graphical display of the mean rating among 2 full-time study staff when evaluating the importance of certain preparatory, recruitment, and data collection strategies to the success of the survey participation and completion across 31 hospitals and 72 nursing homes**. Rating was performed on a scale of 1 to 4, with 1 being unimportant, 2 being somewhat important, 3 being very important, and 4 being critical to success. Intra-rater reliability was significant with 64% of ratings being identical and 96% being either identical or in an adjacent rating level. DPH refers to the Department of Public Health. IRB refers to Institutional Review Board.

Two full-time staff members were responsible for all logistical aspects of this project. They prospectively evaluated the value of specific recruitment and data collection strategies for all 31 hospitals and 72 nursing homes who were approached to participate(Figure 1). Among preparatory strategies, the collaboration with the Orange County Health Care Agency provided access to lists of facility and laboratory contacts, and increased the visibility and perceived legitimacy of the study through the use of Orange County Health Care Agency badges and logos. This was a major advantage when scheduling face-to-face meetings and ultimately recruiting participants. In addition, detailed training of study staff on hospital epidemiology and healthcare-associated infection content was critical for attaching credibility and importance to the surveys.

### Strategies Contributing to Successful Recruitment

Among recruitment strategies, four things contributed to a high participation rate (Figure 1). The first was to ensure that all initial recruitment conversations occurred in a face-to-face meeting. The second was persistence, which was provided in the following ways: 1) serial phone calls for initial contact, 2) presentations to initial contacts who would authorize subsequent presentations to final decision-makers, 3) contacting higher-level decision-makers when initial contacts were unsuccessful, and 4) repeated presentations to maintain recruitment due to high administrative turnover. Multiple recruitment presentations were required to secure the participation of 55%, 26%, 40%, and 36% of administrators, infection control programs, laboratories, and nursing homes respectively (Table 2). Notably, 10 hospitals and eight nursing homes required three or more presentations for the Administrative Survey.

**Table 2:** Healthcare Survey Recruitment Presentations to Orange County Hospitals and Nursing Homes

	Number of Presentations		
	1	2	3+
**Acute Care Facilities**			
Administrative Survey (N = 29)^a^	13	6	10
Infection Control Survey (N = 27)^b^	20	6	1
Microbiology Survey (N = 16)	10	5	1
**Nursing Homes**			
Administrative Survey (N = 72)	43	18	8

^a ^Two facilities declined an in person presentation

^b ^Two Infection Control and Prevention programs covered 2 hospitals each.

The third factor was our use of healthcare facility social networks, which enabled higher levels of participation. Enthusiastic participants encouraged close colleagues in similar positions from other facilities. Existing monthly meetings and social networks of local chapters of healthcare organizations (Association for Professionals in Infection Control and Epidemiology, California Association of Health Facilities) furthered opportunities for such networking. Finally, confidentiality was considered critical to our success. Facility and patient confidentiality concerns were expressed by several facilities despite the fact that all surveys only requested summary-level facility information. Despite lack of any patient-level data or identifiers in any survey, study staff members were required to obtain institutional review board approval from 5 participating hospitals and file a formal application for institutional review board exemption for one hospital. None of the 65 nursing homes required institutional review board approval. Study staff members were also required to sign HIPAA privacy statements at 30% of participating hospitals.

### Strategies for Data Receipt

**Figure 2:**
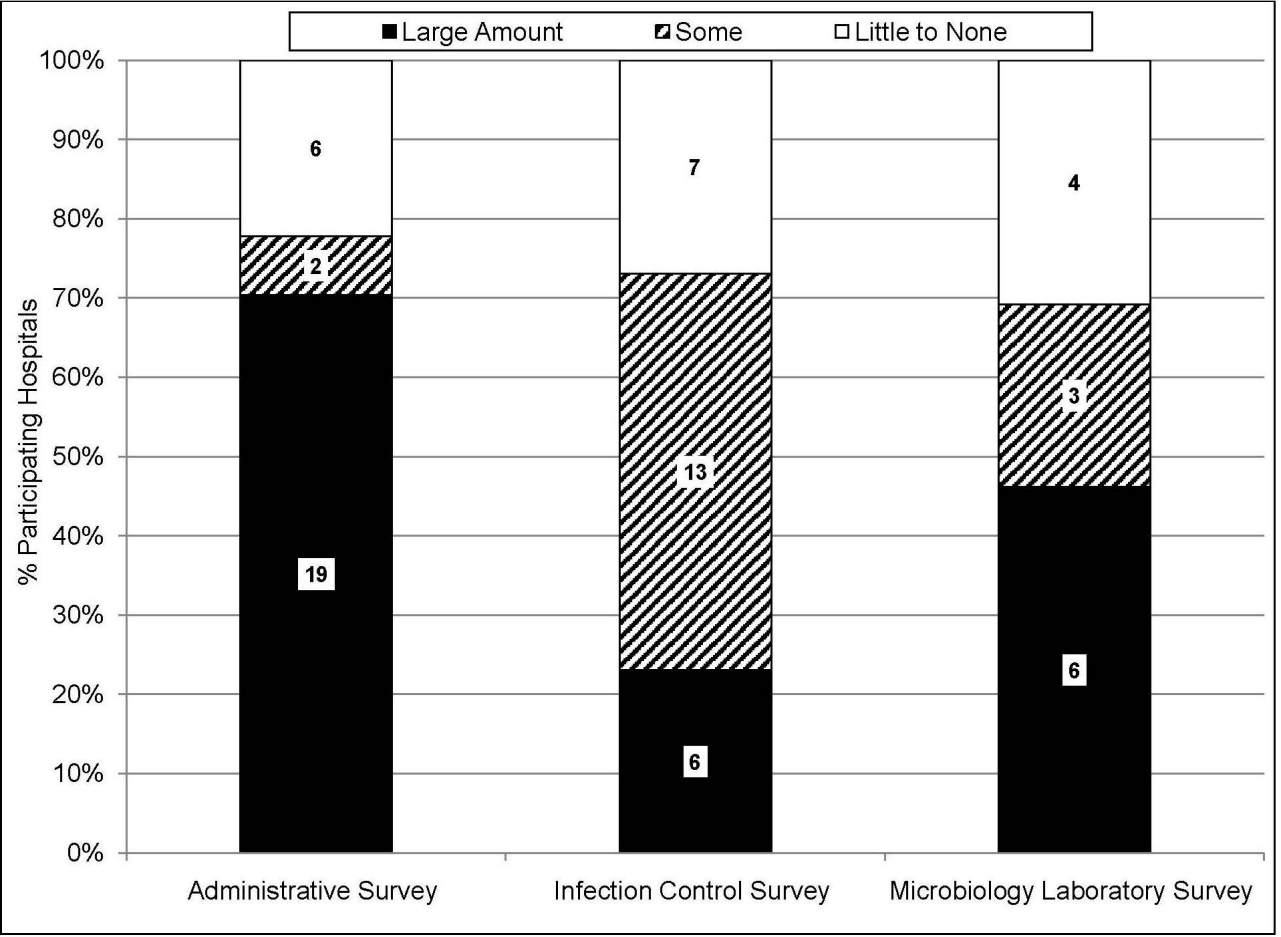
**Bar graph depicting the fraction of participating hospitals which required provision of study staff labor in order to complete the survey**. Labor mostly included summary-level tallying of information located in paper files, as well as performing chart reviews for limited data elements. Black color indicates a large amount of labor was needed, hatched bars indicate some labor was needed, and white color indicates little to no labor was required.

Due to the request for detailed data tables, provision of labor and flexibility in data collection was necessary for a high rate of data return (Figure 1). The presence of an electronic medical record system in some facilities greatly enhanced the ease and feasibility of completing quantitative data tables. Nevertheless, most facilities did not have such systems. Of the facilities that returned data, participation was contingent on the provision of staff labor in 21(78%) hospitals participating in the Administrative Survey, 19(73%) hospitals participating in the Infection Control Survey, and 9(69%) laboratories participating in the Microbiology Laboratory Survey (Figure 2). Common activities included tabulating case counts from electronic or paper reports, and performing chart reviews for limited pre-specified data elements. Labor would not have been necessary if surveys had not requested data tables.

In addition, flexibility was essential. Project staff allowed data to be collected from a recent 12-month period even if months were not completely contiguous, and prospective data was accepted. Reasons for noncontiguous data included: 1) data capture issues due to change over to electronic medical record systems, 2) temporary failure of electronic data storage programs, 3) lack of surveillance data collection due to temporary vacancy of infection control positions. Furthermore, although facilities were urged to complete the survey within four weeks, latitude was given to encourage participation. Major reasons for delays included hospital inspections by The Joint Commission or the California Department of Public Health, new state mandatory reporting requirements, and administrative turnover and vacations. Nursing homes were generally more approachable due to the smaller number of administrative staff. In addition, since their annual volume was substantially lower than hospitals, they needed considerably less project staff labor or flexibility in data collection. However, nursing homes still required a comparable level of persistence to secure participation and timely data receipt.

### Time to Recruitment

**Figure 3:**
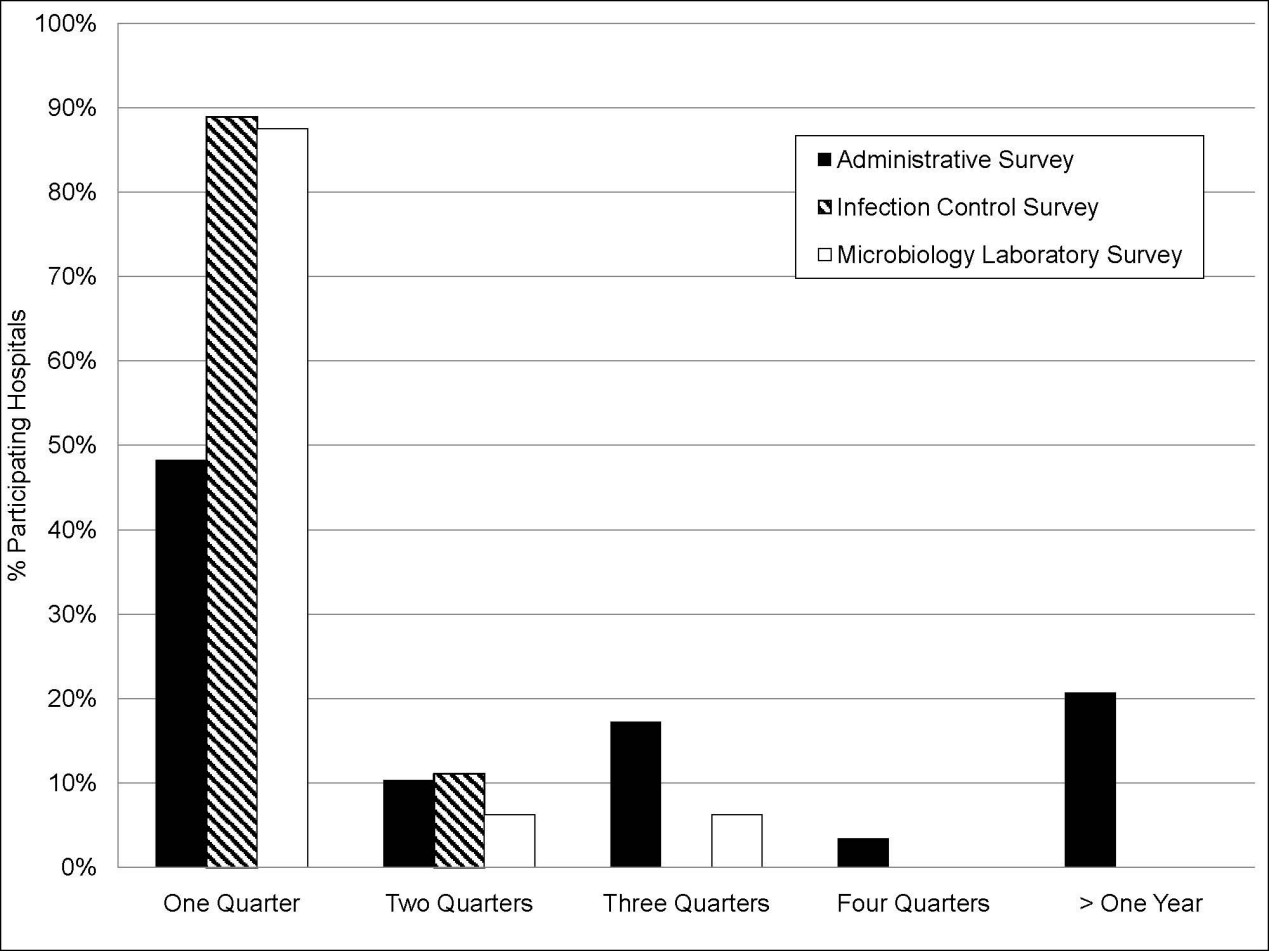
**Graphical display of the recruitment time in quarters required to secure hospital participation in facility-level surveys**. Black bars indicate percent of hospitals participating in the Administrative Survey (N = 29); hatched bars, the Infection Control Survey (N = 27); and white bars, the Microbiology Laboratory Survey (N = 16 laboratories serving 31 hospitals).

The amount of time required to recruit hospitals varied widely(Figure 3). For the Administrative, Infection Control, and Microbiology Laboratory surveys, respectively, 48%, 89%, and 88% of participants were recruited within one quarter, and 58%, 99%, and 94% within two quarters. Nursing homes, due in part to smaller size and detailed logbooks, required considerably less labor assistance to complete the Administrative Survey compared to hospitals (33%(20/61) vs 78%(21/27), p = 0.001). Among hospitals, delay was caused by administrative hurdles (e.g. IRB approval, difficulty scheduling recruitment meetings), or the need for repeat presentations due to administrative turnover(Table 2). Among nursing homes, 68% were recruited within one quarter, with 83% recruited within two quarters. The major delay in nursing homes was due to the high frequency of administrative turnover.

### Time to Data Receipt

**Figure 4:**
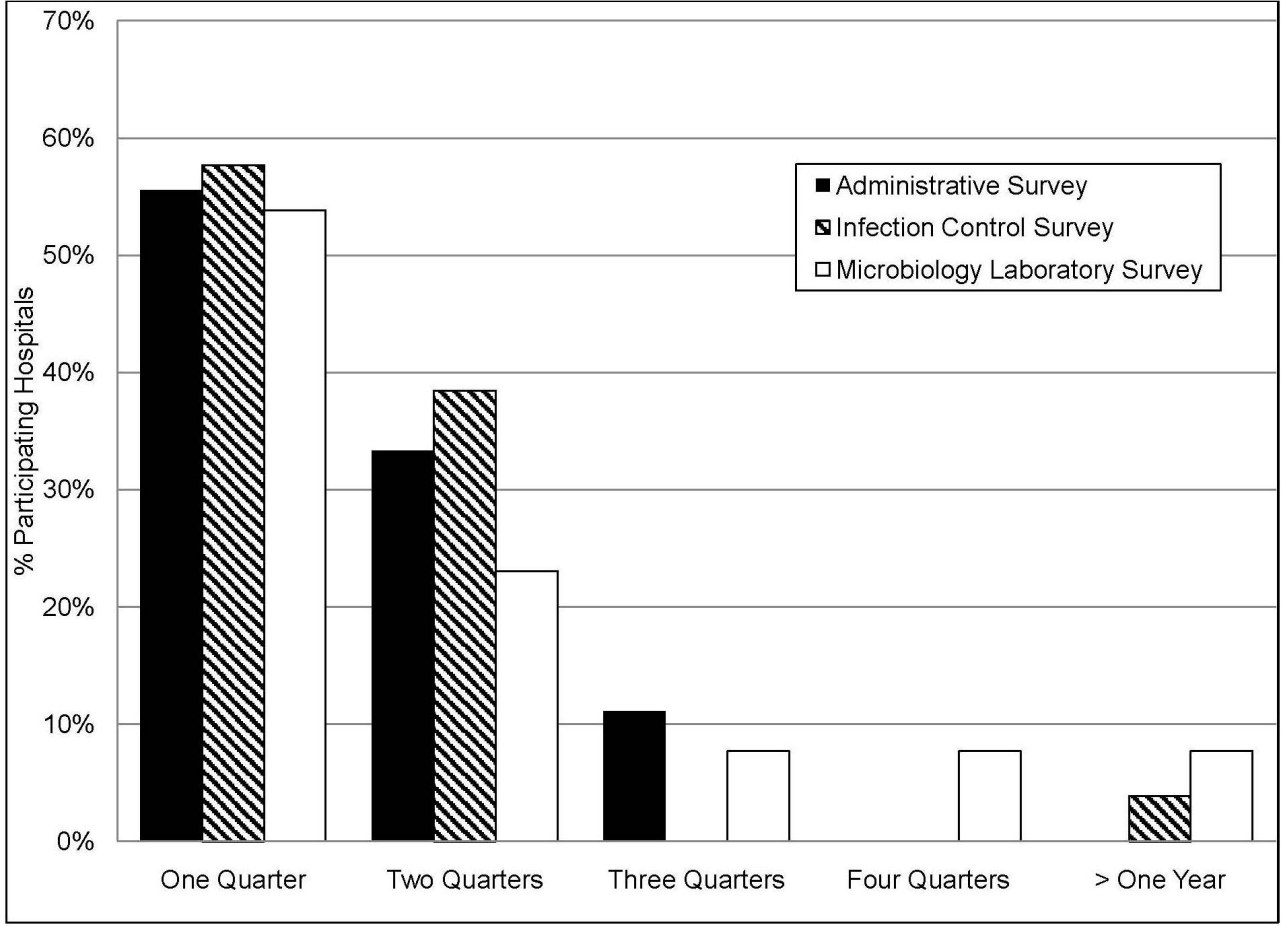
**Graphical display of the time in quarters from a hospital's agreement to participate in the survey until survey completion**. Percentages of hospital participants are shown based upon the survey type, including the Administrative Survey (black bars, N = 27), Infection Control Survey (hatched bars, N = 26), and Microbiology Laboratory Survey (white bars, N = 13 laboratories serving 31 hospitals). Of those agreeing to participate, 2 hospitals did not complete the Administrative Survey, 1 hospital did not complete the Infection Control Survey, and 3 laboratories did not complete the Microbiology Laboratory Survey.

Of the facilities that provided data, 89% of Administrative Surveys, 96% of Infection Control Surveys, and 77% of Microbiology Laboratory Surveys were returned within 6 months (Figure 4). Similar to recruitment, project staff encountered delays due to accreditation surveys, electronic data extraction issues, and labor requirements. Because of provided labor assistance, the percent of received Administrative Survey data was similar for nursing homes and hospitals (85%(52/61) vs. 89%(24/27), p = 0.2) within 6 months from agreement to participate.

### Data Completeness

**Figure 5:**
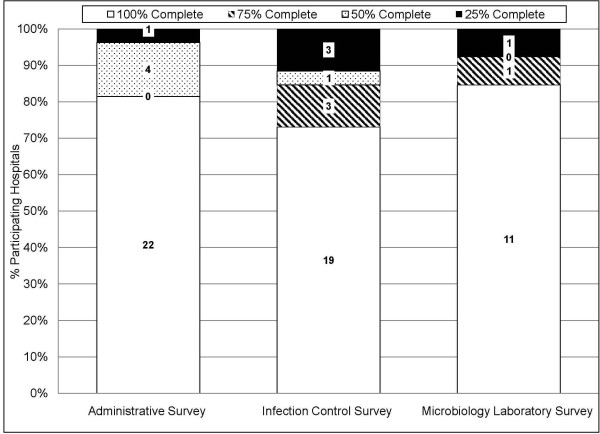
**Bar graph depicting the degree of survey completeness by survey type**. White bars indicate surveys were 100% completed; hatched bars, 75% completed; dotted bars, 50% completed; and black bars, 25% completed. Missing or non-existent data, electronic data limitations, and prohibitively large labor requirements were responsible for the return of incomplete surveys.

Despite offers of assistance, 5/27(19%) Administrative Surveys, 7/26(27%) Infection Control Surveys, and 2/13(15%) Microbiology Laboratory Surveys were returned with at least some incomplete data elements(Figure 5). On the contrary, 90% of nursing homes provided 100% complete data. Missing or non-existent data, electronic data limitations, and prohibitively large labor requirements were responsible for the return of incomplete surveys.

As with any undertaking, there were strategies that proved unsuccessful or provided little benefit compared to the time and effort invested. When making initial contact, efforts were best spent on scheduling an in-person meeting. Hospital administrators who asked for project details over the phone often found the ensuing information daunting. Providing details in person with the survey in hand proved to be the most fruitful for the project. During in-person meetings, the recruitment staff could read body language, tone, and facial expressions in order to better engage and recruit facilities. Second, while latitude in time to data provision increased total data return it also resulted in considerable elongation of the study, which may not be possible or desirable in other circumstances.

## Discussion

We share our experience with implementing three regional healthcare surveys and describe potentially generalizable strategies for garnering high participation. Healthcare facility based surveys to assess standardized reporting of healthcare-associated infection patient outcomes are becoming more important as public reporting becomes required by legislative mandates [1,9,16]. Regional surveys are particularly helpful to understand practices or disease prevalence that might be geographically based. While incentives for high compliance participation is understood for surveys of individuals, far less is known about the factors that enable surveys of institutions. [14,15]. The intent of this paper is to make transparent our experience in conducting regional hospital and nursing home surveys and to reveal obstacles to obtaining high participation. As others do the same, the collective of published experience in conducting regional surveys, albeit anecdotal, can facilitate serial learning by preventing others from repeating past mistakes and allowing them to capitalize on the previously vetted successful strategies of others.

This growing requirement for hospitals and nursing homes to report performance measures to improve patient safety or disclose incidence of healthcare-associated infection has increased the need for accurate population-based comparisons. We undertook these three large regional surveys to assess whether standardized definitions were being used for measures at risk for future reporting. Our intent was to define regional benchmarks and determine whether or not non-uniform definitions exist. This type of survey introduced two levels of complexity. First, high regional participation was needed to ensure data was not biased by high performing facilities that may be more willing to share data. This need for high participation led to the provision of substantial flexibility in time to recruitment and data receipt.

Second, the request for detailed data tables imposed the need to offer study staff labor to gather summary level counts from available line lists, reports, or even medical records. Without this offer of labor, over half of the hospitals and one quarter of the nursing homes would not have participated, and participation would have heavily favored facilities with an electronic medical record system and the ability to tabulate data. Not surprisingly, less resourced hospitals required more study staff labor, such that exclusion due to lack of labor could have introduced bias if worse outcomes were related to poor resources. The national incentives for electronic medical records under meaningful use guidance will likely facilitate these types of healthcare surveys in the future [17]. This is equally true for efforts to create direct laboratory reporting to public health departments, regional healthcare information exchanges, and distributed electronic health data networks [18-20]. In the absence of readily accessible data, reasonable compliance in a shorter amount of time may be garnered if data tables are not required and only moderate participation is needed across targeted facilities.

In addition, the new era of quality measurement centered around inter-facility comparison has ushered in a spirit of competitiveness and vulnerability that hampers healthcare surveys even though they may be devoid of patient-level health information. Had we not guaranteed facility-level confidentiality to our participants, we would have had zero participants because of our request for healthcare-associated infection rates and acquisition rates of multi-drug resistant organisms, despite the fact that these measures are current and future targets for public reporting. Explicit written and verbal assurance of facility-level confidentiality in our study protocols was required by several hospital administrators before they would agree to participate.

Furthermore, we highlight the value of partnering with the local department of public health. This provided a trusted and recognized name to the project and greatly facilitated scheduling recruitment meetings with facility administrators by ensuring attention to and consideration of the request for participation. Interestingly, because of the focus of our surveys, once an in-person meeting was secured many facilities then required assurance that data would remain confidential and would not be shared with the divisions of public agencies that are involved with either accreditation surveys or state or public reporting.

Each of the above strategies would have been considerably less effective had they not been coupled with consistent use of in-person meetings and study staff who were dedicated to maintaining a positive rapport with regional facilities. While garnering participation was a major goal of individual visits, it was of greater importance to create, foster, and maintain strong relationships with each of the key contacts because of the broader impact that a disgruntled facility might have on regional participation. The amount of inter-facility discussion and relationships should not be underestimated in a single geographic area. Maintaining relationships proved to be invaluable since participants often influenced other departments within the same facility, encouraged participation by administrators at other facilities, and even provided avenues for increasing project visibility through invited speaking engagements. This was all the more important because administrative turnover frequently resulted in personnel changes, often from one regional facility to another.

Prior researchers conducting assessments of healthcare facilities have not provided full descriptions or assessments of their survey methods [21-24]. Nevertheless, Jones et al. achieved 87% participation in an anonymous survey of hospitals with whom they had an existing relationship [21]. Another brief survey of hospitals by the New York State Department of Health had a return rate of 99%, presumably highlighting the influence that the Health Department involvement can have on survey participation [24]. This study also used connections with local infection control associations and extensive follow up. A third study surveyed hospitals owned by a single corporation, and used the corporate infrastructure to garner a return rate of 80% for a questionnaire requiring provision of quantitative data, as well as answering multiple choice and free response questions [23]. While it is not possible for us to know which strategies produced the high participation rates in these surveys, we note that they used some of the same strategies we describe here.

## Conclusions

Regional healthcare surveys are important for benchmarking and assessing standardized data collection for health outcomes in the era of mandatory reporting. We found that partnership with the local county health department, persistence in recruitment, latitude in time to data receipt, and provision of substantial study staff labor were valuable elements in ensuring very high participation in healthcare surveys directed at regional hospitals and nursing homes, which combined qualitative and quantitative elements. We further found that the assurance of facility-level confidentiality was imperative to several administrators given our interest in health outcomes that would reflect overall facility safety and performance. This descriptive study provides detailed examples of the required effort and strategies behind a high participation countywide survey of healthcare facilities in order to help others with similar research or public health endeavors.

## Competing interests

The authors declare that they have no competing interests.

## Authors' contributions

KE assisted with study design, data collection and analysis, and drafted the manuscript. CN assisted with the study design and data collection. DK was responsible for data cleaning and assisted with data analysis and manuscript drafting. HM assisted with recruitment and survey design. MC assisted with recruitment and survey design. SH conceived the project obtained funding, designed the study, and oversaw the analysis. All authors read and approved the manuscript.

## Author's Information

SH is currently an Associate Professor in the Division of Infectious Diseases and the Health Policy Research Institute at the University of California Irvine School of Medicine. She also serves as the Medical Director of Epidemiology and Infection Prevention at the University of California Irvine Medical Center.

HM is the Medical Director of the Department of Epidemiology and Assessment at the Orange County Healthcare Agency. Her contributions in this joint collaboration were instrumental in garnering support from the hospitals and nursing homes throughout Orange County.

MC is the Associate Medical Director of the Department of Epidemiology at the Orange County Healthcare Agency. Her contributions in this joint collaboration were instrumental in garnering support from the hospitals and nursing homes throughout Orange County.

## Pre-publication history

The pre-publication history for this paper can be accessed here:

http://www.biomedcentral.com/1471-2288/11/176/prepub
